# Experimental assessment of antidiarrheal and antisecretory activity of 80% methanolic leaf extract of *Zehneria scabra* in mice

**DOI:** 10.1186/1472-6882-14-460

**Published:** 2014-12-02

**Authors:** Wondmagegn Tamiru Tadesse, Abebe Ejigu Hailu, Abyot Endale Gurmu, Abraham Fikru Mechesso

**Affiliations:** Department of Pharmacology and Clinical Pharmacy, School of Pharmacy, Addis Ababa University, Addis Ababa, Ethiopia; Unit of Pharmacognosy, School of Pharmacy, University of Gondar, Gondar, Ethiopia; School of Veterinary Medicine, Hawassa University, Hawassa, Ethiopia

**Keywords:** Anti-diarrhea, *Zehneria scabra*, Anti-secretory, Rodents

## Abstract

**Background:**

The leaf of *Zehneria scabra* is traditionally used for the management of diarrhea in Ethiopia. Its use, however, has not been scientifically validated for safety and efficacy. The aim of this study was to investigate antidiarrheal and antisecretory effects of hydroalcolic leaf extract of *Z. scabra* in mice models.

**Methods:**

For each of antidiarrheal, gastrointestinal motility and antisecretory activity study Swiss albino mice were divided in to five groups. Group I was treated as control group and received 10 ml/kg of 2% Tween-80 orally; Group II served as a positive control and took standard drug in each of the experiments orally; Group III, IV and V were test groups which received the methanolic extract orally at 100, 200 and 400 mg/kg, respectively. Depending on the model total weight of fecal output, total weight of wet feces, total number of fecal output, number of wet faeces, length of intestinal transit and intestinal weight were collected. Finally, data were analyzed using one-way ANOVA followed by Tukey’s post test.

**Result:**

In castor oil induced diarrhea model, the extract dose produced a significant reduction in mean stool score (1.94 ± 0.102) at 200 mg/kg. Moreover, the 100, 200, and 400 mg/kg doses inhibited stool frequency by 40, 45 and 55%, respectively. All test doses of extract and loperamide (3mg/kg) reduced fecal fluid content significantly (p<0.01). The 100 mg/kg dose of extract produced 25.74% reduction of fluid content (p<0.001) while both 200 and 400 mg/kg showed 29.70 % (p<0.001) compared to negative control group.

**Conclusion:**

The extract of *Zehineria scabra* showed antidiarrheal and antisecretory activity in mice model. Moreover, the extract found to be safe at dose of 2000mg/kg in mice model. The findings suggest the validity of the acclaimed effect of *Zehineria scabra* as antidiarrheal agent in Ethiopian traditional herbal medicine.

## Background

Diarrhea is the passage of abnormal liquid or unformed stool at an increased frequency. Infectious agents, certain medications, plant and animal toxins, gastro-intestinal disorders, and substances that increase gastrointestinal tract secretions may cause it. It can also be caused by the ingestion of poorly absorbable materials, or inflammatory and dysmotility problems of the gastro-intestinal tract [1–3].

Diarrheal diseases are one of the leading causes of morbidity and mortality in developing countries and are responsible for the death of millions of people each year. There are a number of epidemiological and experimental evidences worldwide related to acute diarrheal disease, which is one of the principal causes of death in the infants [4]. Around 2.5 million children die each year worldwide and 80% of which are reported in developing countries. Diarrhea is most common in crowded living conditions coupled with poor hygiene and malnutrition [5].

Although currently used drugs are important in the management of diarrhea, they still are linked with adverse effects and contraindications. For instance, racecadotril and loperamide are used to treat secretary diarrhea but they produce bronchospasm, vomiting and fever. Moreover, some are contraindicated in children below 6 years of age (loperamide) and intestinal obstruction. Different antibiotics used do have valuable importance to fight this condition; however, drug resistance is another issue to think about [6].

Despite immense technological advancement in modern medicine, many people in the developing countries still rely on healing practices and medicinal plants for their health care needs. Moreover, to combat the problem of diarrhea in developing countries, the World health organization (WHO) has constituted diarrhea control program aimed at a holistic approach to include all aspects of traditional medical practices, evaluation of health education and preventive approaches [7]. A range of medicinal plants with antidiarrheal properties has been widely used by the traditional healers; however, the effectiveness of many of these anti-diarrheal traditional medicines has not been scientifically evaluated [8].

Traditional medicine practice has been documented in Ethiopia for treatment of various ailments. Including diarrhea for which by various plants has been used. *Zehneria scabra* (Linn.f.) Sond. (Cucurbitacea) (vernacular name - ‘hareg ressa’) is one of the commonly used medicinal plants in Ethiopian traditional medicine practices. Being a climbing or trailing herb, it can go up to 10 m in length. Stems become woody with corky-ridged bark as they grow old. And it has ovate, broadly ovate or pentagonal leaves. Traditionally in Ethiopia the flowers of the plant has reportedly been used for topical treatment of alopecia, wound and eczema along with other herbals mixed together [9]. Additionally, the leaves [10], fruit, and flower have been used for the treatment of abdominal colic in decoction of water and taken orally [11]. The traditional use of the leaves of this plant for the treatment of diarrhea is also reported in rural central Ethiopia and Burundi besides its use for skin reaction [11]. Scientific works to testify to any of the claims are almost non-existent except a few studies that conducted antimicrobial activity test for the various extracts of the dried, powdered leaves of *Z. scabra.* The results were promising with the aqueous and methanolic extracts exhibiting interesting inhibitory activity against *S. aureus*, *P. aeruginosa*, *E. coli* and *C. albicans* [12, 13]. Related to phytochemical studies, NMR analysis of the ethyl acetate root extract of the plant reported the presence of gypenoside group, like many other Cucurbitacea family members [14].

It is necessary to establish scientific evidences for therapeutic use of such traditional medicinal plants, as it may potentially be useful source of new effective therapies in drug development process. Hence, this study aimed at evaluating the traditional claim of antidiarrheal and antisecretory effect of methanolic leaf extract of *Zehneria scabra* and its acute toxicity in mice model.

## Method

### Drugs and chemicals

Methanol (RANKEM, India), Tween-80 (Sigma-Aldrich, UK), Activated Charcoal (Lab. Reagent, India), Castor Oil and loperamide were obtained from a local retail outlet in Addis Ababa. Atropine Sulfate (AdvaCare, USA), Hydrochloric Acid (BDH Ltd, England) Chloroform (ACS, Merck), Sulfuric Acid (Farm Italia Carrloerba, Italy), Ammonia (Merck Millipore, India), Acetic Anhydride (Techno Pharmchem, India), Ferric Chloride (Fisher, USA), Potassium Ferrocyanide (BDH Ltd, England), Lead Acetate (BDH Ltd, England), and Ethyl Acetate (ACS, Merck).

### Plant material

The leaves of *Zehneria scabra* were collected from Debre Tabor and surrounding area. Taxonomic identifications were then established by the Department of Biology, National Herbarium, Addis Ababa University. A voucher specimen was also kept there (Voucher sample number WT/03).

### Experimental animals

Healthy male Swiss albino mice, weighing between 20-30g, were obtained from animal house of Pharmacology department, University of Gondar. The animals were housed in a polyethylene glycol cage (5 to 8 animals per cage) and acclimatized, for a minimum of 5 days prior to pharmacological studies. The animals were maintained under standard condition of relative humidity (44-56%), temperature (25 ± 2°C) and 12/12 h light–dark cycle [15, 16] and allowed free access to pellets and tap water.

### Extraction procedure

The leaves of *Zehneria scabra* were collected and thoroughly washed with distilled water to remove dirt and soil. The plant material was air-dried at room temperature and pulverized into a dry powder. Then, 100 g of this material was macerated with 500 ml initially and then the residue was macerated with 400 ml and 300 ml of 80% methanol, successively. The respective extract was filtered using gauze and Whatman No-1 filter paper, and dried under reduced pressure, at temperature below 45°C in oven. The percentage yield of the process was 17.5% (w/w). The extract material was kept in an air-tight container and stored at 4°C until needed. The extract was semisolid at room temperature and solidified at storage in a refrigerator. When re-exposed to room temperature, however, the extract turned in to semisolid.

### Preliminary phytochemical screening test

The crude extract was screened for the presence or absence of secondary metabolites such as alkaloids, steroidal compounds, phenolic compounds, saponins, tannins and anthraquinones using standard procedures by coloring and precipitation assays using standard procedures as described elsewhere [17, 18].

### Acute toxicity test

Acute toxicity test was done based on the limit test recommendations of OECD 425 Guideline [16]. First, female Swiss albino mouse was fasted for 4 h and then loaded with 2000 mg/kg of the extract, orally. The mouse was then observed for physical or behavioral changes within 24 h strictly, with special attention during the first 4 h. Based on the results found from the first mouse, other four female mice were recruited and fasted for 4 h. Then, the animals were administered a single dose of 2000 mg/kg followed by similar strict observation. Such observation was continued for further 14 days for any signs of toxicity.

### Grouping and dosing of animals

The animals were randomly assigned to different groups- as negative control group, treatment groups for different doses of extracts of *Zehneria scabra*, and positive control group. Group I was negative control and was given a vehicle, (2% (v/v) Tween-80 in water). Group II (positive control group) was given standard drug, loperamide (3 mg/kg, orally) in both castor oil induced antidiahreal test and misoprostol induced antisecretory test and atropine sulfate (0.1 mg/kg, i.p.) for gastrointestinal motility test by charcoal meal. Group III to V (test groups) were given the extract of *Zehneria scabra* at doses of 100 (ZS100), 200 (ZS200) and 400 (ZS400) mg/kg, respectively. Plant extracts and the standard drug, loperamide were dissolved with distilled water, were administered orally and volume administered was 10 ml/kg for all animals.

As per OECD [16] guideline, after the safety evaluation of the plant, 1/10^th^ of the maximum dose (2000 mg/kg) was considered as a middle dose, half and double of the middle dose were taken as the low and the high doses, respectively. The test doses were prepared freshly on the day of the experiment. Animals were treated with vehicle or treatment 1 h before subjecting to the different tests.

### Anti-diarrhea activity tests

#### Antidiarrhea activity on Castor oil-induced diarrhea

The method described by Franca 2008 [19] was followed for this study. The animals were screened initially by giving 0.5 ml of castor oil and only those showing diarrhea were selected for the final experiment. Each animal was placed in individual cage, the floor of which was lined with blotting paper and every hour the floor lining was changed. Diarrhea was induced by oral administration of 0.5 ml castor oil to each mouse, 30 min before the above treatments. Every hour, total weight of faecal output, total weight of wet faeces, total number of faecal output, and number of wet faeces were recorded. A numerical score based on stool consistency was assigned as follows: normal stool = 1, semi-solid stool = 2 and watery stool = 3 [20]. And % inhibition of diarrhea was calculated as follows:


The *in vivo* antidiarrheal index (ADI *in vivo*) was then expressed according to the formula [21]:


Where D_DT_ is the delay in defecation time or diarrheal onset (as % of control), G_MT_ is the Gasrointesitinal motility by charcoal travel reduction (as % of control), and N_FS_ is the reduction in the number (frequency) of stools (as % of control).

### Gastrointestinal motility test by charcoal meal

After the grouping, each animal was loaded with 1 ml of charcoal meal (3% deactivated charcoal in normal saline) orally. After 30 min of respective treatment of each group, the animals were sacrificed and the movement of charcoal from pylorus to cecum was measured. The charcoal movement was expressed in terms of percentage [22]. The percentage of inhibition of gastrointestinal motility was determined by using the following equation:


### Antisecretory assay

The effect of the hydroalcoholic extract on fluid secretion in intestine, which was induced by misoprostol at 20 μg/kg, was studied using mouse model. Mice were fasted for 24 h before induction by misoprostol. 1h later each group was then treated just as stated above in the grouping and dosing of animals. Mice were then sacrificed after 24 h and fluid accumulation ratio (the weight of intestine to the rest of the body weight of mouse) was recorded and the antisecretory activity was expressed in percentage of inhibition [23].


In addition to this, the weight of wet stool and dry stool (after 24 hr of defecation) were used to calculate the fluid content in the stool and the difference between wet stool and dry stool was recorded as stool fluid content.

### Statistical analysis

All data were expressed in appropriate data presentation methods. Statistical analysis was carried out using one way ANOVA followed by Tukey’s post hoc tests. The results were considered significant at p-value less than 0.05. For data processing and analysis SPSS statistical software Version 20.0 was used.

### Ethical clearance

All procedures were complied with The Guide for the Care and Use of Laboratory Animals [15] and OECD-guideline-425 for acute toxicity [16]. The procedures were approved by the Institutional Review Board of the college of medical and health sciences, University of Gondar and ethical clearance was obtained from the research and publication office of the University (Ref. No. CMHS-RPO/045/2013).

## Results

### Preliminary phytochemical screening

The leaves of *Z. scabra* crude extract revealed the presence of various secondary metabolites based on preliminary phytochemical screening test (Table 1). Among the detected in the crude extract were tannins, saponins, anthraquinones, O-anthraquinones and phenols. On the other hand, alkaloids and steroidal compounds were not detected.

**Table 1:** **Phytochemical screening results of 80% methanolic extract of leaves of** ***Zehneria scabra***

Test	Result
Alkaloids	-
Tannins	+
Saponins	+
Anthraquinon glycosides	+
O-anthraquinones	+
Phlabotanins	+
Steroidal compounds	-
Phenolic compounds	+

+: present, -: absent.

### Acute toxicity test

None of the animals showed behavioral, neurological or physical changes characterized by symptoms such as reduced motor activity, restlessness, convulsions, coma, diarrhea and lacrimation at the limit dose of 2000mg/kg of the hydroalcoholic extract of *Z. scabra* during the observation period. In addition, no mortality was observed at the test dose. Thus, the median lethal dose (LD50) of the plant extract was found to be greater than 2000 mg/kg.

### The effect of *Z. scabra*extract on castor oil-induced diarrhea in mice

The effect of the extract on the mean stool score and number of wet feces is shown in Table 2. After 30 min of the administration of castor oil, all the animals of the negative control group (2% TW-80 treated) were apparently diarrheic with a mean stool score of 2.68 ± 0.05 out of 3 scale stool score. *Z. scabra* extract at doses 100 mg/kg (p<0.05), 200 mg/kg (p<0.001) and 400 mg/kg (p<0.01) significantly reduced the mean stool score and the number of wet feces (p<0.001 for all doses) compared to the negative control. Whilst loperamide significantly decreased mean stool score and number of wet feces compared to controls (p<0.001), no statistically significant difference was noted when compared with the other doses of the extract. It is of note that maximum reduction was achieved still with loperamide (84.5%).

**Table 2:** **The effect of** ***Z. scabra*** **extract on stool score scale in castor oil- induced diarrhea in mice**

Group	Onset of diarrhea	Mean stool score	Number of wet feces	% Inhibition of diarrhea
2%TW-80	66.4±3.06	2.68 ± 0.05^a^	19.4 ± 2.44^a^	-
Lop3	180.5±15.45	1.49 ± 0.093***	3 ± 0.89***	84.5
ZS100	100.33±8.41	2.34 ± 0.098*^a^	9.3 ± 1.20***^b^	52.1
ZS200	118.33±7.34	1.94 ± 0.102***^b^	7 ± 0.85***	63.9
ZS400	140.00±16.48	2.26 ± 0.119**^a^	6.5 ± 0.81***	66.5

Values are mean ± S.E (n=5) ***p<0.001 values compared with the negative control; **p<0.01 values compared with the negative control, *p<0.05 values compared with the negative control. ^a^p<0.001 values compared with the positive control; ^b^p<0.01 values compared with the positive control.

Moreover, treatment with *Z.scabra* produced a similar pattern of change in number of defecation as depicted in Table 3. Accordingly, a significant reduction in the number of defecations over four hours and the total number of defecation was observed with all the test doses of the extract compared with the vehicle treated group. All test doses demonstrated significant reduction in number of defecation starting from the 2^nd^ hour through the fourth hour with percent inhibition of defecation 40, 45 and 55% for 100, 200, and 400 mg/kg doses, respectively. However, no apparent change was noted among the different doses of the extract. The standard drug also showed a marked reduction in number of defecation by about 65% compared to vehicle treated group, which was by far the highest of all. However, once again no detectable changes were noted when compared to all doses of the extract.

**Table 3:** **The effect of** ***Z. scabra*** **extract frequency of defecation on castor oil-induced diarrhea within four hours in mice**

Group	Number of deifications in specific time				Total number of defecation	Inhibition (%)
	1hr	2hr	3hr	4hr		
2%TW-80	4.6 ± 2.86	9.4 ± 1.12^a^	4.2 ± 0.8^a^	1.8 ± 0.49^b^	20 ± 2.43^a^	-
Lop3	5.2 ± 1.14	1.8 ± 0.70***	0 ± 0.00***	0 ± 0.00*	7 ± 1.57***	65
ZS100	4.5 ± 0.72	4.2 ± 1.01**	2 ± 0.37*^b^	1.5 ± 0.56^b^	12 ± 0.95**	40
ZS200	6.3 ± 0.615	2.7 ± 0.42***	1.7 ± 0.50**	1 ± 0.31	11 ± 0.43**	45
ZS400	5.7 ± 1.33	1.7 ± 0.21***	1.2 ± 0.40**	0.5 ± 0.22	9 ± 1.53***	55

Values are mean number of defecations ± S.E (n=5), *p< 0.05 compared with the negative control, **p< 0.01 compared with the negative control, ***p< 0.001 compared with the negative control. ^a^p<0.001 values compared with the positive control; ^b^p<0.01 values compared with the positive control.

Stool fluid content is calculated as the difference of the weight of wet stool (fresh) to that of the dried (measured after 24 h of defecation). As demonstrated in the Table 4, treatment with 100 mg/kg and 200 mg/kg of the extract did not produce significant decrease in stool fluid content in the 1 h, 2 h and 4h compared to negative control. By contrast, 400 mg/kg of the extract did show significant reduction (p<0.05) in the stool fluid content by 0.05 ± 0.02 at 2 h. At the 3 h, all doses of the extract and the positive control loperamide (3 mg/kg) treated groups demonstrated a significant difference of fluid content with 0.00 ± 0.00 (p<0.001), 0.09 ± 0.03 (p<0.01), 0.04 ± 0.02 (p<0.001) and 0.05 ± 0.02 g (p<0.001) compared to that of 0.29 ± 0.08 g of the negative control group. However, there was no significant difference in stool fluid content at the 4 h among the groups.

**Table 4:** **The effect of** ***Z. scabra*** **on weight composition of wet and dried stool in castor induced-diarrhea in mice**

Group	Stool fluid content in Weight (g)			
	1hr	2hr	3hr	4hr
2%TW-80	0.18 **±** 0.12	0.50 **±** 0.04	0.29 **±** 0.08)^a^	0.04 **±** 0.03
Lop3	0.07 **±** 0.09	0.36 **±** 0.18	0.00 **±** 0.00***	0.01 **±** 0.01
ZS100	0.26 **±** 0.10	0.28 **±** 0.05	0.09 **±** 0.03**	0.04 **±** 0.02
ZS200	0.40 **±** 0.09	0.18 **±** 0.07	0.04 **±** 0.02***	0.07 **±** 0.04
ZS400	0.42 **±** 0.11	0.05 **±** 0.02*	0.05 **±** 0.02***	0.01 **±** 0.01

Values are mean weight of stool ± S.E. (n=5), *p< 0.05 compared with the negative control, **p<0.01 compared with the negative control, ***p<0.001 compared with the negative control. ^a^p<0.001 values compared with the postive control.

### The effect of *Z. scabra*extract on interstitial motility in mice

The effect of *Z. scabra* extract on the intestinal transit is depicted in Table 5. All doses of the extract failed to produce significant alteration in the % intestinal motility compared to the negative control. The negative control (2% Tween-80 solution) resulted in 90.6 ± 9.2% intestinal motility by the marker-charcoal meal. The 100, 200 and 400 mg/kg oral dose of the extract exhibited 86.9 ± 8.4, 86.1 ± 5.9% and 78.0 ± 9.4% intestinal motility, respectively (Table 5). However, the standard drug, atropine demonstrated a significant inhibition in intestinal motility.

**Table 5:** **The effect of** ***Z. scabra*** **extract on intestinal transit in mice using charcoal meal as a marker**

Group	Total length of intestine (cm)	Distance travelled by charcoal (cm)	% Intestinal transit	% Inhibition relative to control
2%TW-80	59.2 ± 1.48)	53.7 ± 6.41^a^	90.6 ± 9.2^a^	-
Atropine	57.1 ± 2.86	0 ± 0.0	0.0 ± 0.0	100
ZS100	56.6 ± 5.86	49.1 ± 6.0^a^	86.9 ± 8.4^a^	4.1
ZS200	59.9 ± 4.793	51.6 ± 2.9^a^	86.1 ± 5.9^a^	4.9
ZS400	55.8 ± 6.80	43.8 ± 9.8^a^	78.0 ± 9.4^a^	13.9

Values are mean length of intestine ± S.E (n=5). ^a^ p<0.001 values compared with the positive control.

### The effect of *Z. scabra*extract on intestinal fluid secretion by weight ratio

As demonstrated in Table 6, all test doses of the extract significantly reduced the intestinal weight ratio in dose dependent manner. The 100 mg/kg oral dose of the extract produced 25.74% inhibition of weight of intestinal content (p<0.001) relative to 2% TW-80 treated negative control group. Moreover, both the 200 and 400 mg/kg treated groups showed 29.70% inhibition of intestinal weight ratio (p<0.001) relative to the negative control group. In comparison to loperamide (3 mg/kg) treated group, all tested doses of the extract depicted superior percentage of reduction in weight of intestinal ratio.

**Table 6:** **The effect of** ***Z. scabra*** **extract on fluid secretion in Misoprostol-induced mice using small intestine to the body ratio**

Group	Weight of small intestine (g)	Ratio of small intestine to the rest body of mice	Inhibition of intestinal content (%)
2%TW-80	2.09 ± 0.20	0.101 ± 0.012^a^	-
Lop3	1.56 ± 0.28	0.076 ± 0.006*	24.75
ZS100	1.58 ± 0.26	0.075 ± 0.006*	25.74
ZS200	1.41 ± 0.19	0.071 ± 0.004*	29.70
ZS400	1.17 ± 0.37	0.071 ± 0.009*	29.70

Values are mean ± S.E (n=5), *p< 0.001 compared with the negative control. ^a^p<0.001 values compared with the positive control.

### In vivo antidiarraheal index

Results for the *in vivo* antidiarheal index were 17.33, 21.31 and 34.25 at the dose of 100, 200 and 400 mg/kg oral doses of the plant extract, while loperamide gave a maximum index of 74.33.

## Discussion

Several studies have validated the use of antidiarrheal medicinal plants by investigating the biological activity of extracts of such plants, which have antispasmodic effects, delay intestinal transit, suppress gut motility, stimulate water adsorption, or reduce the intraluminal fluid accumulation [23]. The leaves of Z*. scabra* have been used for its antidiahreal effect in Ethiopian folk medicine without any scientific validation for its safety and efficacy [24, 25]. However, it is evidenced to have antimicrobial activity against *E.coli, S.auerus, P.aueroginosa and C. albicans* [12, 13] *.* The present study was conducted to validate antidiarrheal efficacy of Z*. scabra* extract in the different experimental models of diarrhea. In the present study the methanolic extract of Z*. scabra* have showed antidiarrheal activity in different animal models.

Despite the multiplicity of etiologies of diarrhea, literatures state that there are four major pathophysiologies that lead to diarrhea. These include increased luminal osmolarity (osmotic diarrhea), increased electrolytes secretion (secretory diarrhea), decreased electrolytes absorption, and deranged intestinal motility causing a decreased transit time [26]. As an intervention of diarrhea, many anti-diarrheal agents elicit effects by reducing the gastrointestinal motility and/or the secretions [23].

In contrast, laxatives and diarrhea causing agents enhance gastrointestinal motility and/or secretions. For instance, castor oil; which is used as an inducer of diarrhea in this study, is known for its laxative effects because of the active principle, retinoic acid. The active principle of castor oil is known to change the electrolyte permeability of the intestinal membrane and through elevated prostaglandin biosynthesis and release it causes diarrhea similar to pathophysiologic conditions that cause diarrhea [27, 28]. Because of this, castor oil was used to induce diarrhea in the experimental animals of this study. Different researches have shown that castor oil causes diarrhea 1–2 h just after administration of 0.1-0.3 ml for mice [29]. In our experiment diarrhea response was seen within 1 h in most of the experimental subjects because of the high dose of castor oil (0.5 ml/mice). Only those mice that showed the diarrheal response were selected for the experiment, to evaluate the effects of *Z. scabra* leaf extract.

The hydroalcolic extract of *Z. scabra* (100–400 mg/kg, p.o.) significantly (p <0.001) reduced the fecal output produced by castor oil at each hour interval and, of course, in the overall 4h interval. At doses of 100–400 mg/kg (p.o.), the plant extract significantly (p < 0.001) and dose dependently delayed the onset of diarrhea induced by castor oil when compared with the untreated controls. *Z. scabra* (200 mg/kg, p.o.) reduced the number of fecal episodes by 45% while the dose of 400 mg/kg (p.o.) significantly (p < 0.001) reduced the number of defecation by 55%. However, the percent inhibition by the 100 mg/kg was lower than the 200 and 400 mg/kg, indicating higher dose of the crude extract do have a much better antidiarrheal effect. This might be because it is a sub-threshold (sub-effective) dose. This implies that a relatively high dose of the extract is needed to produce an antidiarrheal. This is in line with other reports of other species of plants in which extracts of plants shown to exert antidiarrheal effect at higher doses [17].

Similarly, the extract demonstrated a significant reduction of mean stool score that indicated change of stool consistency from watery to solid stool. At the same time, the number of wet defecations decreased pointing that the extract demonstrated antidiarrheal effect. 200 and 400 mg/kg doses of the extract were superior in reducing mean stool score and number of wet stools, respectively.

Meanwhile, the standard drug showed a marked reduction in both fecal outputs produced by castor oil and mean stool score by about 65% and 84%, respectively. This was by far the highest of all treatment doses of the extract. This can be attributed to the fact that loperamide exerts its antidiarrheal activity by different mechanisms like regulating the gastrointestinal tract, slowing down motility in the intestine, reducing colon flow rates and consequently any effect on colonic motility [30].

Clinically, diarrhea may result from disturbed bowel function, in which case there is impaired intestinal absorption, excessive intestinal secretion of water and electrolytes, and rapid bowel transit [31]. The antidiarrheal index (ADI) is a measure of the combined effects of these different components of diarrhea such as defecation frequency and onset of diarrheal stools, as well as intestinal motility. The plant extract produced a dose-dependent antidiarrheal index, although its greatest effect was lower than that produced by loperamide, the positive control.

The mean ratio of the weight of small intestine to rest of the body of mice and stool fluid content were used to determine the antisecretory activity of the extract. Consistent with the findings of castor oil induced antidiarrheal model, both parameters at all doses level of the extract demonstrated significant antisecretory effect of the extract. Therefore, it can be deduced that the extract possessed comparable antisecretory activity to the standard drug. Although the experimental antidiarrheal models are not a complete predictors of clinical effectiveness, the findings of this study clearly indicated the antidiarrheal potentials of the plant extract.

The plant extract demonstrated significant reductions of water contents, frequency of defecation and intestinal fluid accumulation. On the other hand, gastrointestinal motility test indicated its poor antimotility effect. The overall findings show that the extract demonstrated a comparable antidiarrheal and antisecretory effects. Although the specific mechanisms of action of the extract need to be explored, the antidiarrheal effect of the extract could be related to inhibition of secretion, reducing intraluminal fluid accumulation or enhancing water absorption but not delaying motility. It is widely reported that different antidiarrheal agents exert their effect through different mechanisms such as inhibiting secretion, decreasing motility, delaying intestinal transit, reducing intraluminal fluid accumulation or by enhancing water adsorption [20, 30, 32].

Phytochemical screening of the plant extract revealed the presence of tannins, phlabotanins, saponins, anthraquinone glycosides, O-anthraquinones. Previous studies also reported the presence of tannins, unsaturated sterols/triterpens and saponins [25]. Different literatures state that components such as tannins, saponins, steroids, terpenes, alkaloids and flavonoids are responsible for antidiarrheal action of different plant extracts through different mechanisms [33, 34]. From which tannins and flavonoids are suggested to be responsible for antidiarrheal activity by increasing colonic water and electrolyte reabsorption whereas the others are linked with inhibiting intestinal motility. Tannins are best known to decrease the irritability of the bowel thereby reducing peristaltic index [35]. Therefore, antidiarrhea and antisecretory effects of *Z. scabra* could be due to the presence of these phytochemicals. Besides, these effects were proved to be seen in the different models of this study, which suggests that several different constituents of the extract might be involved in different mechanism of actions to produce antidiarrheal and antisecretory effects.

Moreover, the acute toxicity study revealed the safety of the extract at the limit dose of 2000mg/kg in mice. At the test dose, mortality and delayed toxicity were not observed in the 14 days post-treatment period. Likewise, death was not recorded during the acute toxicity test. Therefore, based on the findings, the LD50 value of the 80% methanolic extract of the plant is above 2000 mg/kg. This indicates that the methanolic extract of *Z. scabra* is better tolerated and safe after oral administered.

## Conclusion

The 80% methanolic leaf extract of *Zehineria scabra* showed antidiarrheal activity in animal models. Thus, the findings give the experimental basis to understand the traditional claim of the plant. The 100, 200 and 400 mg/Kg doses of the plant extract demonstrated significant antidiarrheal and antisecrtory effect and the higher dose, 400 mg/kg, showed superior efficacy relative to the other doses. However, the extract did not affect the gastrointestinal motility at any of the study doses. The plant extract was also found to have optimal safety margin based on the limit test at 2000 mg/kg dose level acute toxicity test. Therefore, the plant is potentially useful to develop plant based products after further studies to identify the active principle and the mechanism of action.
